# A longitudinal study of the infant nasopharyngeal microbiota: The effects of age, illness and antibiotic use in a cohort of South East Asian children

**DOI:** 10.1371/journal.pntd.0005975

**Published:** 2017-10-02

**Authors:** Susannah J. Salter, Claudia Turner, Wanitda Watthanaworawit, Marcus C. de Goffau, Josef Wagner, Julian Parkhill, Stephen D. Bentley, David Goldblatt, Francois Nosten, Paul Turner

**Affiliations:** 1 Pathogen Genomics, Wellcome Trust Sanger Institute, Hinxton, United Kingdom; 2 Shoklo Malaria Research Unit, Mahidol-Oxford Tropical Medicine Research Unit, Faculty of Tropical Medicine, Mahidol University, Mae Sot, Thailand; 3 Centre for Tropical Medicine and Global Health, University of Oxford, Oxford, United Kingdom; 4 Great Ormond Street Institute of Child Health, University College London, London, United Kingdom; University of Liverpool, UNITED KINGDOM

## Abstract

A longitudinal study was undertaken in infants living in the Maela refugee camp on the Thailand-Myanmar border between 2007 and 2010. Nasopharyngeal swabs were collected monthly, from birth to 24 months of age, with additional swabs taken if the infant was diagnosed with pneumonia according to WHO clinical criteria. At the time of collection, swabs were cultured for *Streptococcus pneumoniae* and multiple serotype carriage was assessed. The bacterial 16S rRNA gene profiles of 544 swabs from 21 infants were analysed to see how the microbiota changes with age, respiratory infection, antibiotic consumption and pneumococcal acquisition. The nasopharyngeal microbiota is a somewhat homogenous community compared to that of other body sites. In this cohort it is dominated by five taxa: *Moraxella*, *Streptococcus*, *Haemophilus*, *Corynebacterium* and an uncharacterized Flavobacteriaceae taxon of 93% nucleotide similarity to *Ornithobacterium*. Infant age correlates with certain changes in the microbiota across the cohort: *Staphylococcus* and *Corynebacterium* are associated with the first few months of life while *Moraxella* and the uncharacterised Flavobacteriaceae increase in proportional abundance with age. Respiratory illness and antibiotic use often coincide with an unpredictable perturbation of the microbiota that differs from infant to infant and in different illness episodes. The previously described interaction between *Dolosigranulum* and *Streptococcus* was observed in these data. Monthly sampling demonstrates that the nasopharyngeal microbiota is in flux throughout the first two years of life, and that in this refugee camp population the pool of potential bacterial colonisers may be limited.

## Author summary

The nasopharynx hosts a community of microbes that first colonise us during infancy and that changes as we grow. Colonisation with certain species is a risk factor for developing respiratory infections such as pneumonia, while other species can have a protective influence. In this study we use molecular methods to identify the bacteria present in nasopharyngeal swabs taken regularly from children in a refugee camp in Thailand. The microbiota develops with age, with early colonisers such as *Corynebacterium* or *Staphylococcus* being eventually outgrown by *Moraxella* and an uncultured taxon described here as unclassified Flavobacteriaceae I. There is evidence in the cohort of *Streptococcus pneumoniae* being frequently carried and transmitted throughout the first two years of life. We found that the microbiota profiles were not unique or distinguishable between individuals in this study, which is unlike studies in high income, low density populations.

## Introduction

Common bacterial colonisers of the infant nasopharynx include *Staphylococcus aureus*, *Streptococcus pneumoniae*, *Moraxella catarrhalis* and *Haemophilus influenzae* [1]. The juvenile nasopharynx is more heavily colonised than in adults [2] which is a risk factor for pneumonia and other respiratory infections: young children are at higher risk and more likely to die from them [3]. Antibiotic consumption causes some changes in commensal communities which can lead to health problems later on [4] and may [5] or may not [6] increase the bacterial load in infants.

Maela (also known as “Mae La”) is a long-term camp for displaced persons located in rural Northwest Thailand. The Shoklo Malaria Research Unit (SMRU) has been providing medical and obstetric care in this refugee population living on the Thailand-Myanmar border since 1986. The camp has a population of approximately 40,000 displaced persons from Myanmar, predominantly of Karen ethnicity, living in a 4km^2^ area. The SMRU clinical framework provides an opportunity to investigate the nasopharyngeal microbiota of children living in this population: how the bacterial composition develops during infancy and early childhood from frontier species to a more mature and stable community, whether respiratory illness is preceded by a change in microbiota profile, and whether the acquisition of a new serotype of *S*. *pneumoniae* may coincide with changes in the rest of the microbiota.

## Results

### Cohort summary

**Fig 1:**
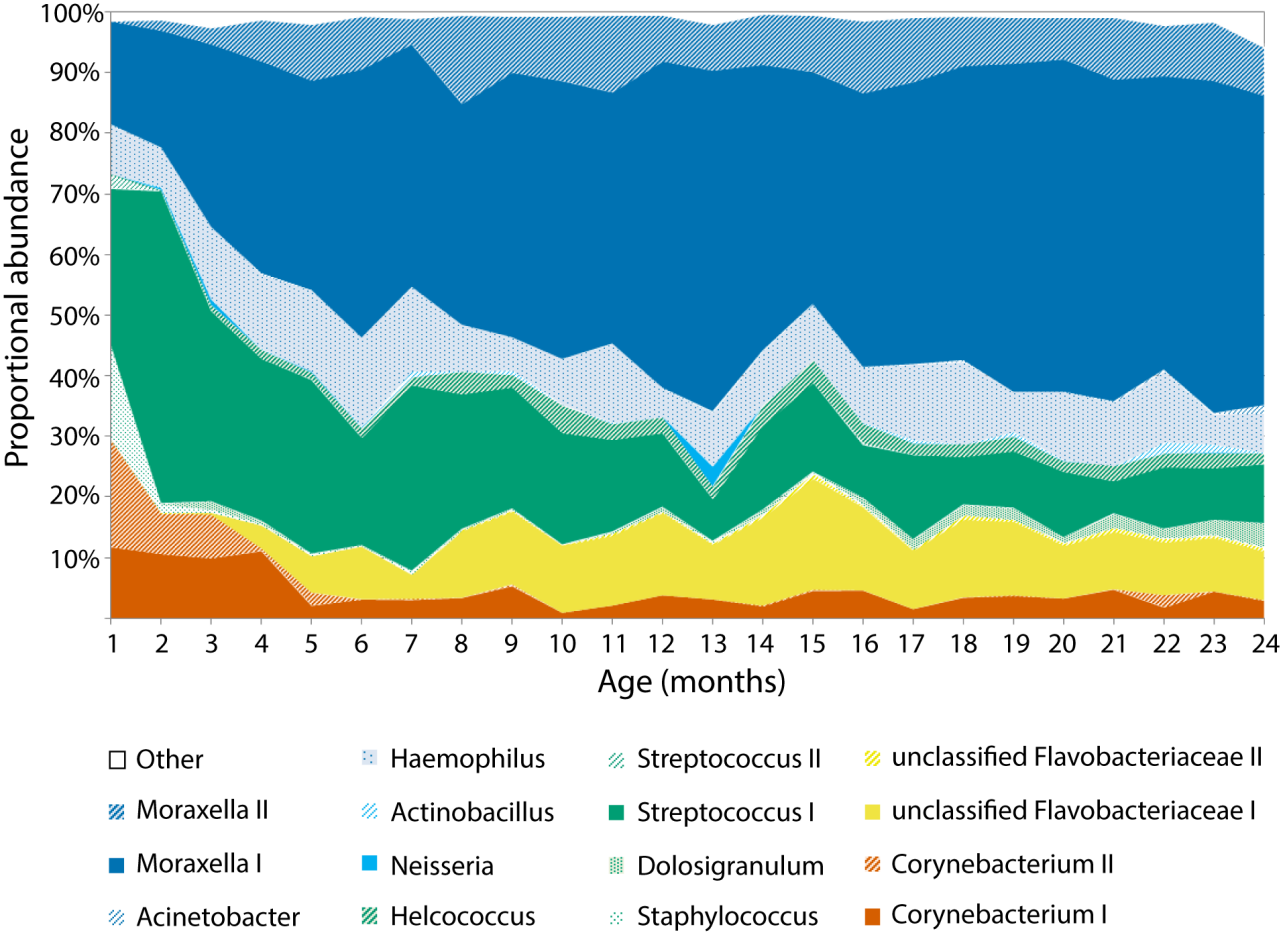
Aggregate of all routine swabs from all infants, proportional abundance of the 15 OTUs that account for >98% of the cohort microbiota, by age in months (1–24). OTUs are colour grouped by phylum: Actinobacteria (orange), Bacteroidetes (yellow), Firmicutes (green), Proteobacteria (blue).

The results are based on rarefied data unless otherwise indicated. 27 samples were lost from the study due to low sequencing depth, leaving 517 samples from 21 infants taken forward for analysis. Data were clustered into operational taxonomic units (OTUs) at a distance of 0.03, of which the most commonly observed were: *Moraxella* I in 98.3% of samples (508/517), *Streptococcus* I in 90.5% of samples (468/517), *Haemophilus* in 77% of samples (398/517), *Moraxella* II in 73.3% of samples (379/517), *Corynebacterium* I in 67.5% of samples (349/517), unclassified Flavobacteriaceae I in 65.2% of samples (337/517) and *Helcococcus* in 62.5% of samples (323/517). Just 15 OTUs account for 98.6% of the aggregated routine microbiota (Fig 1). Metadata and accession numbers for all samples are summarised in Supplementary S1 Table, classification of OTUs in Supplementary S2 Table, and identified contaminants in Supplementary S3 Table.

The median number of OTUs detected in each infant over 24 months is 41 (range 26–91) out of a possible 297. The median sample OTU count per infant is between 5–9 (Supplementary S4 Table). On average each infant experienced 2 episodes of clinical pneumonia during the study, in keeping with previous observations of the Maela infant population experiencing 0.73 episodes per child year [7]. Chest x-rays were interpreted as primary endpoint pneumonia in half of pneumonia episodes, while viral PCR detected respiratory syncytial virus (RSV) in 10/44 samples, human metapneumovirus (HMPV) in 4/44 samples and influenza virus in 1/44 samples (Supplementary S1 Table). No individuals were blood culture positive during illness.

The microbiota of each child is summarised in Supplementary S1 Fig, with pneumococcal serotype carriage indicated alongside. Acquisition of new *S*. *pneumoniae* serotypes is frequent, with as many as 13 different serotypes detected in one child over the study period, but acquisition events do not correlate with the changing proportional abundance of the *Streptococcus* I OTU.

### Changes in the microbiota with age

The infants in this cohort were colonised by bacteria at a very young age: one or more of *S*. *pneumoniae*, *S*. *aureus* or *H*. *influenzae* were cultured from 90% of routine one-month swabs (19/21). In the subsampled dataset illustrated in Supplementary S1 Fig, the *Staphylococcus* OTU was observed in 95% of one-month swabs, *Streptococcus* I in 90%, *Corynebacterium* II in 90%, and *Moraxella* I in 85%.

**Fig 2:**
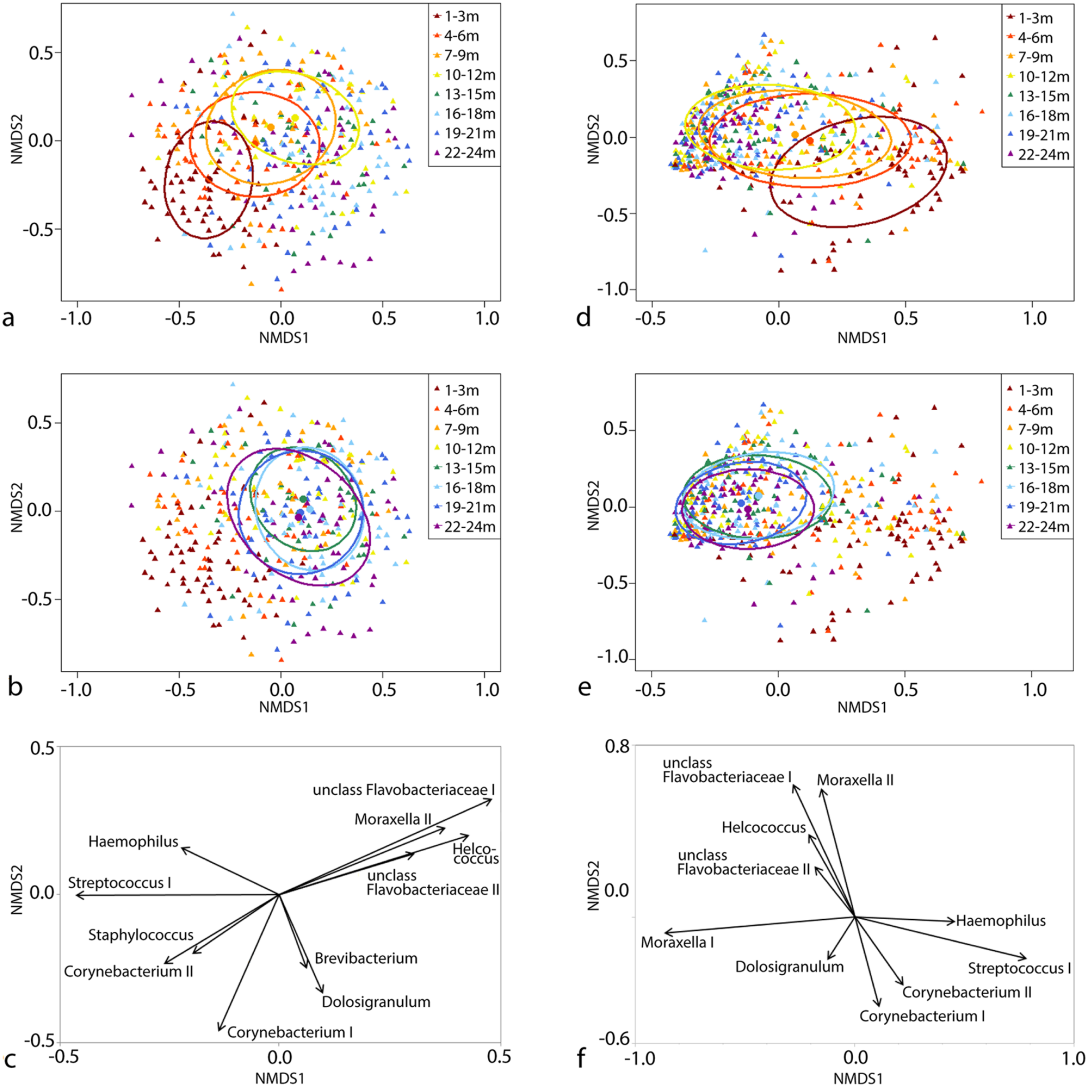
NMDS plot based on Jaccard distance (a-c) and Bray-Curtis dissimilarity (d-f), coloured according to age. a) and d) show 50% ellipses and centroids of the first 12 months of life as an overlay on the full 24-month dataset, while b) and e) show ellipses for 13–24 months overlaid on the full 24-month dataset. Ellipses and datapoints are presented in corresponding colours. Significant OTUs driving the ordination (p<0.0001 and with vector length >0.25) are shown in d) and f). Increasing age is significantly correlated with both x and y axis.

On the whole, shifts in dominance of certain taxa seem to take place from birth to 12 months of age. Analysis of NMDS (non-metric multidimensional scaling) (Fig 2) suggests that although the spread of data is greatly overlapping, there is a trend of changing community composition from birth to 12 months (Fig 2a and 2d) while the samples from 13–24 months show no trend across the graph (Fig 2b and 2d). Increasing age in years correlates significantly with both the x and y axis (Jaccard p = 0 and 0.018 respectively, Bray-Curtis p = 0 and 0.000002 respectively). The most significant OTUs driving the ordination of both Jaccard (Fig 2c) and Bray-Curtis (Fig 2f) are unclassified Flavobacteriaceae I and II, *Corynebacterium* I and II, *Helcococcus*, *Streptococcus* I, *Moraxella* II, *Dolosigranulum*, and *Haemophilus*. The Jaccard ordination was also driven by *Staphylococcus* and *Brevibacterium* while the Bray-Curtis ordination was also driven by *Moraxella* I.

Furthermore, a PERMANOVA test of the Bray-Curtis and Jaccard differences between age groups shows that there is a significant difference (p<0.01) between most non-adjacent age groups, and that 1–3 months samples are significantly different to all other age groups (Supplementary S5 Table).

**Fig 3:**
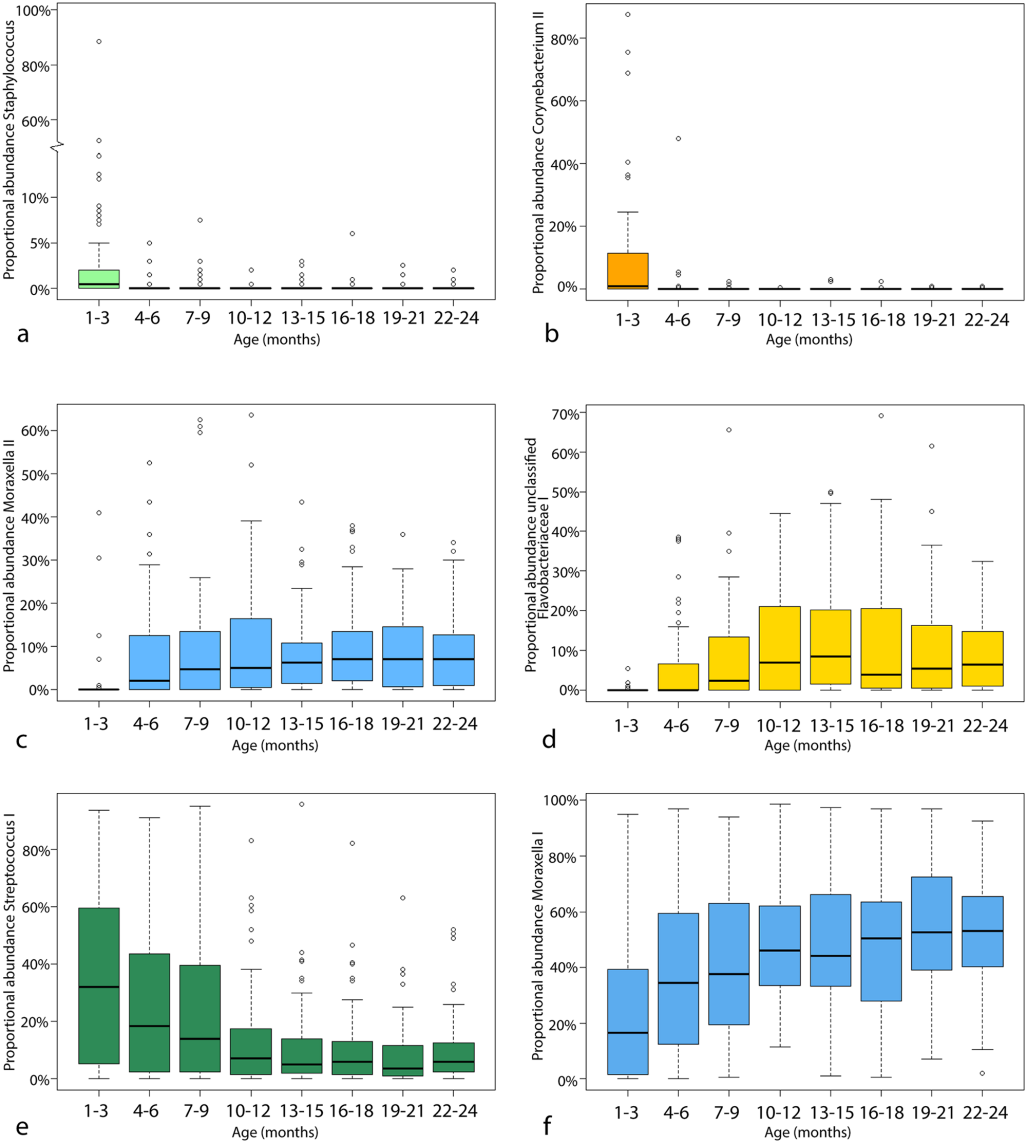
Boxplots of proportional abundances in routine swabs of a) *Staphylococcus*, b) *Corynebacterium* II, c) *Moraxella* II, d) unclassified Flavobacteriaceae I, e) *Streptococcus* I, f) *Moraxella* I.

Several taxa are seen to change with age in the cohort as a whole: *Staphylococcus* and *Corynebacterium* II appear more abundant proportionally in the first 3 months of life (Fig 3a and 3b). In terms of presence/absence (presence defined as 1 read in subsampled data), both the *Staphylococcus* and *Corynebacterium* II OTUs are observed in 78.6% (33/42) of 1–2 month swabs, whereas they are observed in only 16.6% (79/475) and 9% (43/475) respectively of 3–24 month swabs. The median proportional abundance is 4-fold and 9.5-fold higher for *Staphylococcus* and *Corynebacterium* respectively in 1–2 month swabs than older swabs when zero values are excluded, showing that they are not only more commonly observed but also at a higher proportion in younger samples.

*Moraxella* II increases in abundance over the first 9 months and then plateaus (Fig 3c). The unclassified Flavobacteriaceae I rises in average abundance until 12 months (Fig 3d). Conversely *Streptococcus* I decreases in average abundance from 2 months to 12 months and then reaches a stable level (Fig 3e). A longer term pattern is in *Moraxella* I which rises from birth to 21 months (Fig 3f).

Clustering samples based on the 15 most abundant OTUs (Supplementary S2 Fig) provides an alternative view of the age-related patterns observed in Fig 2. The clusters of samples with >50% proportional abundance of *Streptococcus* I, *Corynebacterium* II or *Staphylococcus* are made up of younger samples, whereas the *Moraxella* dominated clusters A1 and D1 are made up of older samples. It is known that early colonisation dominated by *S*. *pneumoniae* is a risk factor for pneumonia illness later in childhood [8], however in this population pneumococcal colonisation rates are high and there are not enough infants to determine whether being in the *Streptococcus* I dominated clusters in early life leads to a greater risk of pneumonia. Similarly there is no evidence that the infants who did not experience any episodes of pneumonia had a similar microbiota profile or belonged to a specific cluster in early life.

### Immunisations

At the time of the cohort study, the WHO EPI-based immunisation schedule in Maela included courses of vaccines against diphtheria, pertussis and tetanus (DTP) as well as viral diseases (measles and polio) and tuberculosis. Neither *Haemophilus influenzae* type b (Hib) nor pneumococcal conjugate vaccines (PCV) were available. The DTP vaccines were given at 6 weeks, 10 weeks and 14 weeks of age. *Corynebacterium diphtheriae* is not observed in the dataset: although this species may fall into the *Corynebacterium* II OTU, it is not detected by oligotyping.

### Microbiology and bacterial 16S rRNA

Culture of target species was positive in fewer swabs overall than by molecular detection. Taking culture to be the gold standard for detection of viable bacteria in a swab and at least one read from the *Staphylococcus*, *Haemophilus* and *Streptococcus* I OTUs in the raw data as a positive molecular detection the sensitivity for *S*. *aureus*, *H*. *influenzae* and *S*. *pneumoniae* was 83.3%, 99.7% and 99.8% respectively. The lower sensitivity of molecular detection for *S*. *aureus* may relate to the lower average proportional abundance of the *Staphylococcus* OTU, because variation between sample aliquots will disproportionately affect detection of the lower abundance species. The specificity however was very poor at 47.4%, 17.2% and 5.8%. This may be due to three issues: that the identity of an OTU is not restricted to a single species, that DNA from lysed or non-viable bacterial cells may be present in the samples, or that the sensitivity of culture itself is not optimal.

### Changes in the microbiota with illness

In this cohort respiratory illness often coincides with a perturbation in the microbiota or heralds a qualitative change in the community going forward, but is different in each child and in different illness episodes. There is no evidence in this study of clinical pneumonia being predicted by a change in the microbiota before illness.

**Fig 4:**
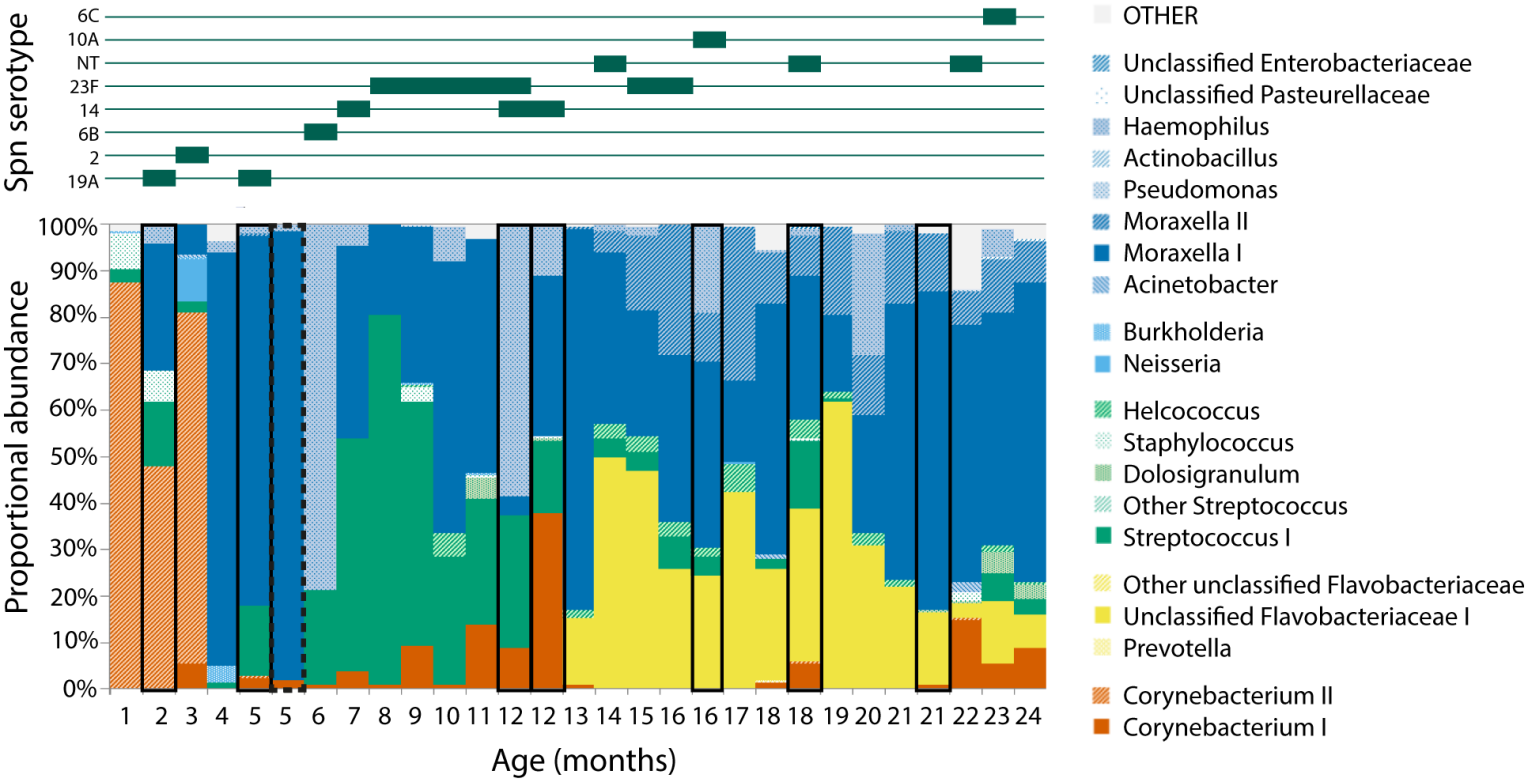
Microbiota profile of one child from 1–24 months of age. Swabs taken during a pneumonia episode are highlighted by boxes. Where cultured, pneumococcal serotype in the sample is indicated in the top pane. OTUs are colour grouped by phylum: Actinobacteria (orange), Bacteroidetes (yellow), Firmicutes (green), Proteobacteria (blue).

For example, infant ARI0218 (Fig 4) had 7 acute respiratory infection (ARI) episodes during the study. Each episode was treated with amoxicillin and the routine swab following the second illness was taken during antibiotic treatment. The first episode at 2 months had little proportional impact on the dominant OTU in the nasopharynx: *Corynebacterium* II. After the second episode the streptococci become dominant, while after the fourth episode the unclassified Flavobacteriaceae OTU becomes dominant.

The inverse Simpson measure of diversity (Supplementary S1 Table) was not found to change predictably when comparing sequential swabs from before pneumonia to the time of clinical presentation: 20 pneumonia episodes coincided with an increase in the diversity measure while 23 appeared to have a decrease in diversity. Nor did it have a consistent pattern comparing samples taken before, during and after antibiotic use. Age was unrelated to whether the diversity increased or decreased in paired samples.

Clustering based on the 15 most abundant OTUs (Supplementary S2 Fig) did not appear to associate with respiratory illness, with the exception of the D8 cluster made up of all the samples with >50% proportional abundance of unclassified Flavobacteriaceae I. These 6 swabs from 5 individuals included 2 samples during pneumonia episodes, 2 taken within 7 days of amoxicillin treatment (pneumonia and tonsillitis respectively), and two routine samples. However cluster D3, with a similar microbiota makeup but a proportional abundance of unclassified Flavobacteriaceae I of 30–50%, had no association with illness (being comprised of 3 ARI and 25 routine samples).

Although clinical pneumonia itself does not seem to have a predictable effect on the microbiota in this cohort, the use of antibiotics may correlate with changing proportional abundance of some taxa. The study included 21 swabs taken during antibiotic consumption and 19 within 7 days of antibiotic use. In most cases the antibiotic consumed was a 7-day course of amoxicillin. Details of the antibiotics in each case are included in Supplementary S1 Table. Tentatively, the distribution of proportional abundance values during antibiotic consumption compared to non-antibiotic samples was lower for *Moraxella* II and higher for *Brachybacterium*, *Dolosigranulum* and *Streptococcus* I (Supplementary S3 Fig). However due to the small number of antibiotic episodes captured in the cohort this pattern could not be reproduced using age-matched samples equally representing each child, and would need to be tested with a larger cross section of samples from the population.

### Interactions between taxa

**Fig 5:**
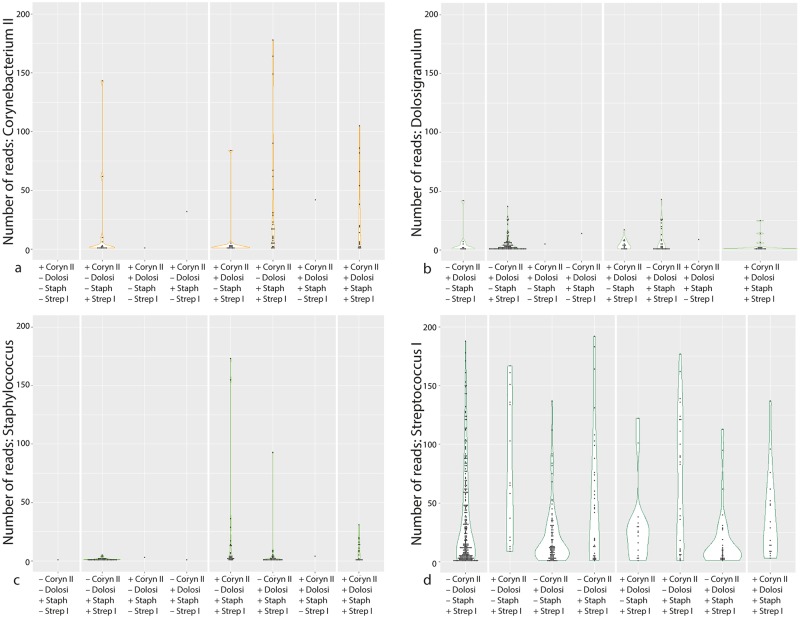
Proportional abundances of a) *Corynebacterium* II, b) *Dolosigranulum*, c) *Staphylococcus*, d) *Streptococcus* I, grouped by the presence/absence of cocolonisers. There is some support for the observation that *Dolosigranulum* is negatively associated with *Streptococcus pneumoniae* in the squat distributions seen in (d) however *Corynebacterium* II does not appear to play a part in that interaction.

It has been demonstrated that there is a complex relationship between *Corynebacterium*, *Dolosigranulum*, *Staphylococcus* and *Streptococcus* in carriage [9]. *Dolosigranulum pigrum* is thought to acidify the environment, creating more favourable conditions for *Corynebacterium* to grow; *Corynebacterium accolens* encourages the growth of *S*. *aureus* while inhibiting *S*. *pneumoniae* through the release of free fatty acids [10]. The negative relationship observed between *Dolosigranulum* / *S*. *pneumoniae* and between *S*. *aureus* / *S*. *pneumoniae* [9, 11] may therefore relate to the *C*. *accolens* intermediary. In this dataset we examined the interacting proportional abundances of four OTUs: *Corynebacterium* II (which may include *C*. *accolens*), *Dolosigranulum*, *Staphylococcus* and *Streptococcus* I (which includes *S*. *pneumoniae*). Samples were grouped according to the presence/absence of these four taxa and the distribution of abundances were compared: both *Corynebacterium* II and *Staphylococcus* appeared to be in higher abundance when they appeared together with *Streptococcus* I (Fig 5a and 5c), while the distribution of *Streptococcus* I abundance was low when it cocolonised with *Dolosigranulum* but it was enriched in the presence of *Corynebacterium* II (Fig 5d). There were not enough datapoints to observe an enrichment of *Staphylococcus* in the presence of *Corynebacterium* II. Although the number of samples is small, these observations do not support the hypothesis that *C*. *accolens* is the true determinant of *S*. *pneumoniae* inhibition because the *Dolosigranulum* signal is inversely correlated with *Streptococcus* I abundance as we expect, but in the absence of *Dolosigranulum Corynebacterium* II presence is positively associated with streptococcal proportional abundance.

Different *Corynebacterium* species are known to interact with *S*. *aureus* in different ways: while *C*. *accolens* promotes the growth of *S*. *aureus*, *C*. *pseudodiphtheriticum* inhibits it [9]. When examining the three OTUs that include these species it does appear that the presence of *Corynebacterium* II (which may represent *C*. *accolens*) presence is associated with a far higher abundance of *S*. *aureus* while *Corynebacterium* I (which may include *C*. *pseudodiphtheriticum*) presence coincides with low abundance. However, the age distribution of *Staphylococcus* and *Corynebacterium* II may be a major confounding factor in this pattern.

We found no link between the age of first colonisation with *Corynebacterium* II or *Dolosigranulum* (in this cohort, on average at 1 month and 4 months respectively) with the number of pneumonia episodes during the study period, although early colonisation with these taxa has been linked to microbiota stability and fewer respiratory infections elsewhere [12].

Despite modelled synergistic and antagonistic interactions between *S*. *pneumoniae* and *H*. *influenzae* [13], no correlation was observed between the proportional abundances of these two OTUs that suggested an influence of one on the other in this cohort. Using cultured samples as a conservative measure, 54% recovered both species (296/544), 24% contained *S*. *pneumoniae* but not *H*. *influenzae* (128/544), 8% contained *H*. *influenzae* but not *S*. *pneumoniae* (44/544), and from 14% of swabs neither species was cultured (76/544).

### A dominant unclassified taxon

Sequences from the unclassified Flavobacteriaceae I OTU share 93% nucleotide similarity to *Ornithobacterium rhinotracheale*, a respiratory pathogen of various wild and domestic birds [14]. They are however >99% identical to an unidentified nasopharyngeal bacterium reported in infants between 4–6 months of age in the Gambia [15], and children aged 1–5 years in Kenya [16]. Oligotyping reveals that there are several subtypes within this group and that an individual tends to be persistently colonised by one oligotype (S4 Fig).

### The personalised microbiota

Some studies have described a wide variation between individuals’ microbiota composition [17] or a “personalised microbiota” [18]. The pairwise Jaccard distances between these samples were compared as in Mika *et al*: at specific ages the distances from the previous and subsequent swabs from the same child (“in”) were compared to the distances to every other child at the same age (“out”). The data from Maela do not resemble the studies in European populations. At each age point the mean in-distances were lower than the out-distances, but the difference was not significant (Supplementary S5 Table) meaning that there is great similarity between the microbial communities carried by cohort members.

### Impact of pneumococcal replacement or acquisition

**Fig 6:**
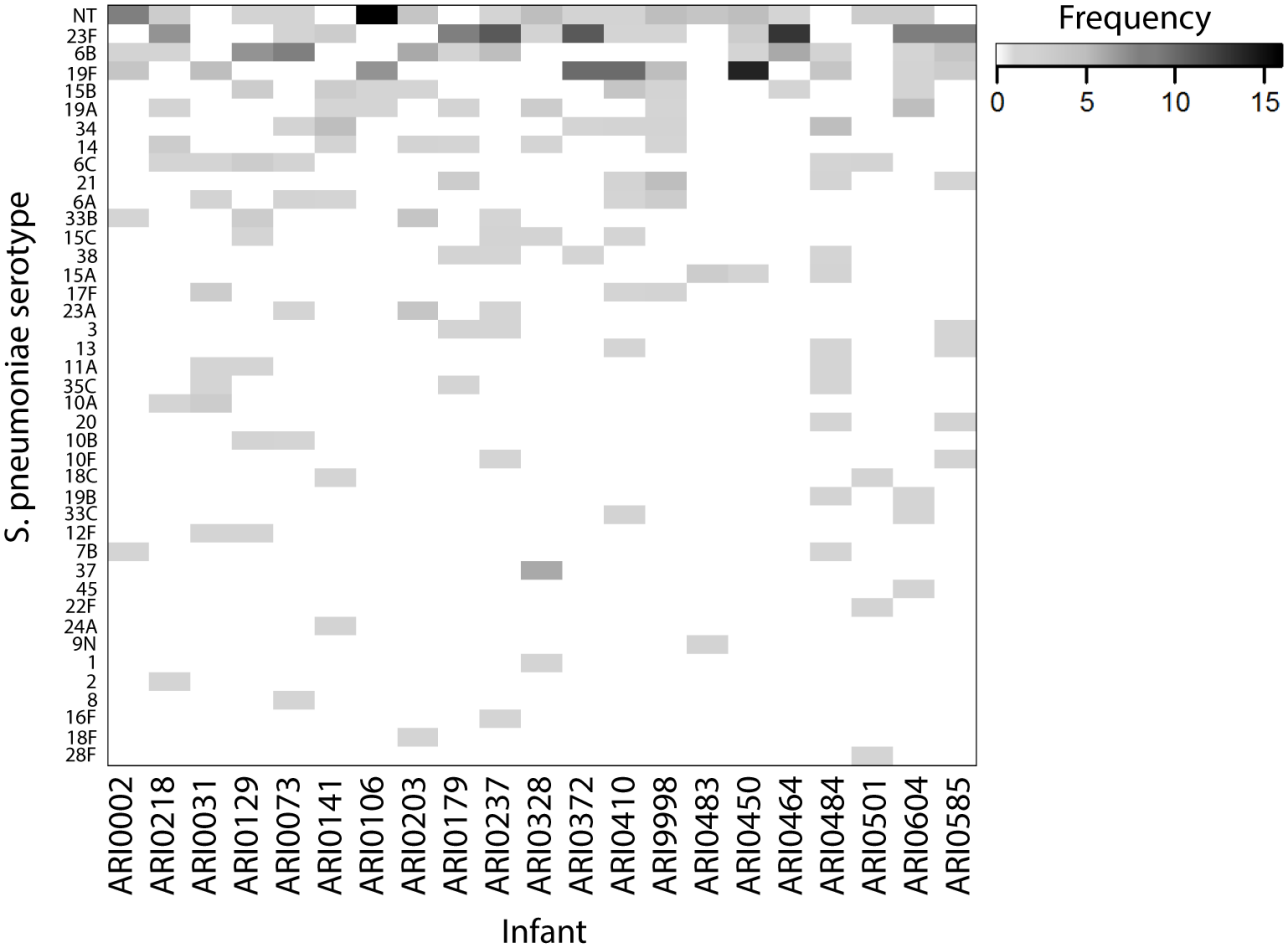
Heatmap of pneumococcal serotypes detected in culture per infant. The mean number of types acquired per child over the study period is 7.4.

During the study, all 21 infants were found to carry *S*. *pneumoniae* at some point: it was detected by culture in 80.9% of swabs (440/544). Multiple serotype carriage was identified in 4.5% of culture positive swabs (20/440). 40 serotypes were identified in total along with non-typeable pneumococci (NT), with a mean of 7.4 different types acquired per infant by 24 months of age. NT pneumococci were cultured in 16 infants (66 samples in total), while the three most frequently observed serotypes were 23F (13 infants, 80 samples), 6B (12 infants, 45 samples) and 19F (10 infants, 64 samples) (Fig 6). Acquisition of a new serotype does not usually coincide with disease or a recognisable shift in proportional abundance of *Streptococcus* I.

## Discussion

The microbiota does change on a cohort level with age (Figs 1 and 3) but it remains somewhat unpredictable from month-to-month (Supplementary S1 Fig) and between infants (Supplementary S5 Fig). Certain taxa are associated with the first three months of life such as *Staphylococcus* (Fig 3a) and *Corynebacterium* II (Fig 3b), while others such as *Streptococcus* I (Fig 3e) decrease gradually over the first year. *Moraxella* (Fig 3c) and unclassified Flavobacteriaceae I (Fig 3d) are rarely observed in 1–3 month swabs and increase in proportional abundance over the first year. These patterns support the hypothesis that there is an early microbial community that is gradually replaced by around one year of age.

A predictable influence from new pneumococcal serotype acquisition or respiratory illness is not supported by these data. The use of antibiotics to treat respiratory infections may affect the microbiota (Supplementary S3 Fig), but the number of observations is too low to draw a robust conclusion. Weather has been shown to play a part in transmission in this population [19] and future work may benefit from taking seasonality into account.

Although the unclassified Flavobacteriaceae I OTU is observed in 65.2% of samples in this cohort and often at high proportional abundance, it has not been successfully cultured here or elsewhere to our knowledge, although unclassified 16S rRNA sequences nearly identical to it have been described in nasopharyngeal samples from the Gambia [15], Kenya [16], and Australia [20]. Further characterisation and genome sequencing of this member of the nasopharyngeal microbiota is being undertaken. For the purposes of identification in 16S rRNA gene datasets, a 642bp consensus from capillary and 454 reads of the oligotype A sequence (the oligotype most frequently found in this study) can be accessed under accession no. LT732570.

This longitudinal study demonstrates the noisy and unsettled nature of the nasopharyngeal microbiota which does not seem to hold a stable individual pattern such as that described in high-income/low density European populations [17, 18]. A hypothetical explanation for this difference between studies is that the increased population density and reduced geographical movement in Maela may lead to exposure to a less diverse pool of potential colonisers, or may simply increase bacterial transmission between children. The results are indicative that single time-point studies may not adequately represent an individual’s microbial community at this age and in a high-density population.

## Materials and methods

### Sample collection

Between 2007 and 2010, a cohort of 955 infants born in the Maela refugee camp on the Thailand-Myanmar border were followed from birth until 24 months of age in a study of pneumococcal colonisation and pneumonia epidemiology [21, 22]. Dacron tipped nasopharyngeal (NP) swabs (Medical Wire & Equipment, Corsham, UK) were collected from each infant at monthly intervals. Immediately following collection, the NP swabs were placed into STGG transport medium (skim milk, tryptone soya broth, glucose glycerol) and were handled in accordance with the WHO pneumococcal colonisation detection protocol [23]. An additional NP swab, plus a NP aspirate for viral PCR [24], was taken if the infant was diagnosed with pneumonia according to WHO clinical criteria [25] and pneumonia episodes were then assessed by chest x-ray. 21 infants who spanned the cohort temporally and who did not have any missing samples, were selected for this study. Sample metadata are described in Supplementary S1 Table. Metadata describing sex, birth weight, mode of delivery, number of rooms in the house, and household members of different age-groups is described in Supplementary S4 Table. All infants were exclusively breastfed, with introduction of solid food at four to six months and cessation of breastfeeding around 1 year of age.

### Ethics statement

Infants were eligible for inclusion in the cohort study if informed written consent had been obtained from the mother during the antenatal period. Participants and their data were anonymised using a 4-digit code prefixed by “ARI-”. The study protocol was reviewed and approved by the ethics committees of the Faculty of Tropical Medicine, Mahidol University, Thailand (MUTM-2009-306) and University of Oxford, UK (OXTREC-031-06).

### Viral PCR, bacterial culture and serotyping

Nasopharyngeal specimens were stored at -80°C within eight hours of collection and subsequently processed in batches. After thawing, 10μL aliquots of homogenised NP swab-STGG specimens were plated onto 5% sheep blood-CNA agar (bioMerieux, Marcy L’Etoile, France) and chocolate agar (Clinical Diagnostics, Bangkok, Thailand). Both plates were incubated overnight at 36°C in 5% CO_2_. *H*. *influenzae*, *M*. *catarrhalis*, *S*. *aureus*, and *S*. *pneumoniae* were identified using standard microbiological techniques. The serotype was determined by latex agglutination for all morphologically distinct pneumococcal colonies, as previously described [26]. Real-time RT-PCR assays were used to determine the presence of influenza virus A/B, respiratory syncytial virus, and human metapneumovirusin the NP aspirates collected during pneumonia episodes.

### DNA extraction and sequencing

Genomic DNA was extracted from a 200ul aliquot of STGG using the FastDNA Spin Kit For Soil (MP Biomedicals, Ohio, USA). Negative controls were also created by processing filtered nuclease-free water (Ambion, Thermo Fisher Scientific, Massachusetts, USA) with each kit batch. Multiplexed bacterial 16S rRNA gene sequencing libraries were created targeting the V3-V4 region with universal primers 338F ([454 adaptor]-ACTCCTACGGGAGGCAGCAG) and 926R ([454 adaptor]-[barcode]-CCGTCAATTCMTTTRAGT) using Accuprime High Fidelity *Taq* DNA polymerase (Invitrogen, Life Technologies, California, USA). Amplification was performed in six 20ul volume PCRs, with the following conditions: 98°C denaturation for 30 seconds, 52°C annealing for 30 seconds, 72°C extension for 2 minutes, with 30 cycles due to low amplicon yield. PCRs were quantified using the Qubit HS DNA assay (Thermo Fisher Scientific, Massachusetts, USA) and in the event of a total yield from a sample of below 30ng, further reactions were performed up to a maximum of 18 reaction volumes (i.e. up to 360ul total volume). These were then pooled, gel extracted to remove primer dimers and sequenced on the GS-FLX Titanium platform (454 Life Sciences/Roche, Connecticut, USA). 454 sequence data is available in the European Nucleotide Archive under accession number ERP020597 and a consensus sequence of capillary and 454 sequence data for the unclassified Flavobacteriaceae I oligotype A is under accession number LT732570. Individual sample accession numbers are listed in Supplementary S1 Table.

### Bacterial 16S rRNA gene analysis

Sequence data for 720 samples (from 544 swabs, with 176 duplicate DNA extractions) and 21 negative controls were analysed using mothur version 1.30.2 [27]. Poor quality reads were discarded (those containing ambiguous bases or homopolymeric tracts longer than 8nt, that had any sequence errors in the primer or barcode, or that were less than 400nt in length after trimming to an average quality score of 35). The dataset was then aligned to a curated SILVA database in order to remove reads that were outside the expected alignment coordinates. Chimeric reads were removed at this stage using chimera.perseus. The dataset was clustered into OTUs at a distance of 0.03 which were classified to the genus level using an RDP reference set [28]. Contaminant and erroneous OTUs were identified as described in the section below. The clean data from duplicate DNA extractions was pooled and subsampled to 200 reads per sample as a compromise between sequencing depth and maintaining continuity of sampling. The rationale behind this strategy is discussed in the Supplementary Methods. Following rarefaction mothur was used to calculate the inverse Simpson diversity score, Jaccard distance and Bray-Curtis dissimilarity, and NMDS ordination. Analysis of “presence/absence” is based upon the presence of one read in subsampled data unless otherwise indicated. Plots, heatmaps, and trees were plotted using R. Boxplots were generated using default settings, specifically with whiskers at the lowest datum within 1.5 interquartile ranges of the lower quartile, and the highest datum within 1.5 interquartile ranges of the higher quartile. PAST3 [29] was used for Bonferroni corrected PERMANOVA (Supplementary S5 Table). Mothur generated high quality reads were used for unsupervised oligotyping “Minimum Entropy Deposition” (MED), which enables fine taxonomic resolution [30]. The oligotype representative sequences were used for species-level classification using ARB software [31] and the SILVA reference library SSURef_123.1_SILVA_03_03_16. The SILVA reference library was manually curated by removing un-cultured and unclassified sequences.

### Contaminant identification and removal

A conservative estimate of contaminant OTUs from extraction kits and other artificial origin was based on a combination of the following criteria, as described previously [32]: 1) presence in negative controls, specifically nuclease-free water passed through the DNA extraction kit and then sequenced alongside the samples. These results were treated with caution due to the appearance of nasopharyngeal taxa in the negative controls as well, possibly due to barcode crossover. 2) Biased or exclusive representation in specific DNA extraction kits, as demonstrated in Supplementary S6 Fig. 3) Mismatch when correlating duplicate DNA extractions. 176 samples were subjected to DNA extraction twice, using different kit lots, allowing some contaminants to be identified by comparing OTU abundance between them. Co-occurrence and independent occurrence of contaminants such as *Ralstonia* could also be observed (Supplementary S6 Fig). 89 further OTUs that could not be classified at the phylum level were discarded as likely to be derived from amplification or sequencing errors. Removed OTUs are detailed in Supplementary S3 Table, while the remaining taxa are described in Supplementary S2 Table.

A number of OTUs were not removed by this process, for example because their abundance was too low to give a strong signal, but their taxonomic identification suggests an environmental origin. These taxa were kept in the analysed dataset but for the reader’s information are indicated in the “contaminant potential” column of Supplementary S2 Table.

## Supporting information

S1 Fig Longitudinal results for the 20 most abundant taxa in the cohort. Where cultured, pneumococcal serotype is indicated above each graph. Solid boxes represent swabs taken at the time of ARI immediately preceding amoxicillin treatment, dashed boxes represent swabs taken during antibiotic treatment. Arrows indicate episodes of non-respiratory disease that required antibiotic treatment. 1a) ARI0031, b) ARI0073, c) ARI0106, d) ARI0129, e) ARI0141, f) ARI0179, g) ARI0203, h) ARI0218, i) ARI0237, j) ARI0328, k) ARI0372, l) ARI00450, m) ARI0410 n) ARI9998 (the twin of ARI0410), o) ARI0464, p) ARI0483, q) ARI0484, r) ARI0501, s) ARI0585, t) ARI0604, u) ARI0002 (diagnosed with tuberculosis during the study).
(PDF)

S2 Fig Heatmap of samples clustered based on the 15 most abundant OTUs. (PNG)

S3 Fig Proportional abundance for routine, illness, antibiotic (during treatment) and post-antibiotic (within 7 days of treatment) swabs for a) Moraxella II, b) Brachybacterium, c) Dolosigranulum, d) Streptococcus I. “Healthy”, “antibiotic” and “post-antibiotic” are samples collected at routine monthly intervals, grouped retrospectively based on the concurrent antibiotic consumption.
(JPG)

S4 Fig Distribution of the three main oligotypes of unclassified Flavobacteriaceae I between infants (samples in order of age, left to right within each box). Within an individual the specific oligotype is persistent over time, suggesting that colonisation is stable and transmission is low for this taxon.
(PNG)

S5 Fig Distribution of pairwise Jaccard distances at seven timepoints: 3, 6, 9, 12, 15, 18 and 21 months of age. “In” distances are within an individual, comparing one month before/after the timepoint. “Out” distances are between individuals of the same age. Pairwise distances within an individual are lower on average than inter-individual distances at every age, but the difference is not significant.
(PNG)

S6 Fig An example of contaminant identification. OTUs may be skewed in distribution to specific kits (left side). The DNA introduced during the extraction process will not correlate in duplicate extractions of the same sample (right side). A kit profile may be observed by certain OTUs being associated with one another (bottom): Otu0019 Ralstonia, and Otu0020 an unclassified taxon that has been identified in a variety of published aquatic studies, these correlate well in kits 1 and 12 but Ralstonia is also present independently in kit 15.
(JPG)

S1 Table Sample metadata, read counts, culture results. (XLSX)

S2 Table Classification of OTUs. (XLSX)

S3 Table Contaminant and removed OTUs. (XLSX)

S4 Table Participant metadata. (XLSX)

S5 Table PERMANOVA (Bonferroni corrected) p-values for the Bray-Curtis and Jaccard differences between pairs of age groups, values <0.01 shaded. Younger age group samples are different to more age groups than the older samples.
(XLSX)

S1 Methods Description of the rationale behind the compromise of subsampling depth and continuity of sampling for these data. (DOCX)

S1 Checklist STROBE checklist. (DOCX)
