# Supplementary figures and images for: A longitudinal study of the infant nasopharyngeal microbiota: The effects of age, illness and antibiotic use in a cohort of South East Asian children
Source: PLoS Negl Trop Dis. 2017 Oct 2;11(10):e0005975. doi: 10.1371/journal.pntd.0005975 (PMC5638608; doi:10.1371/journal.pntd.0005975)

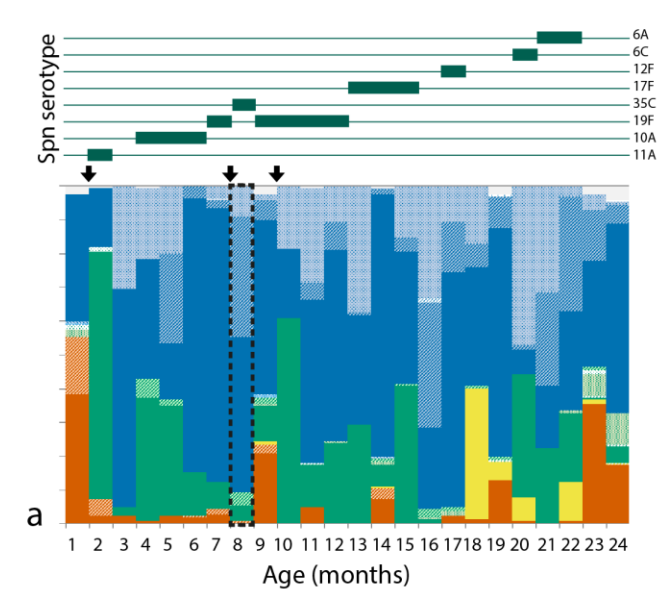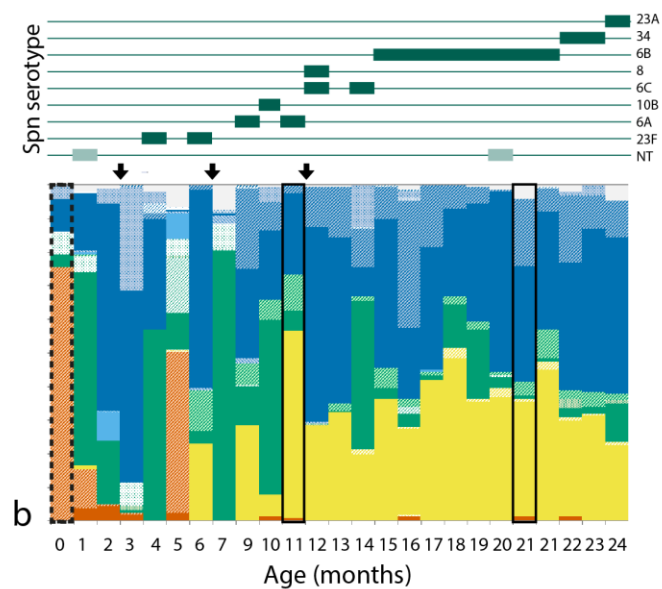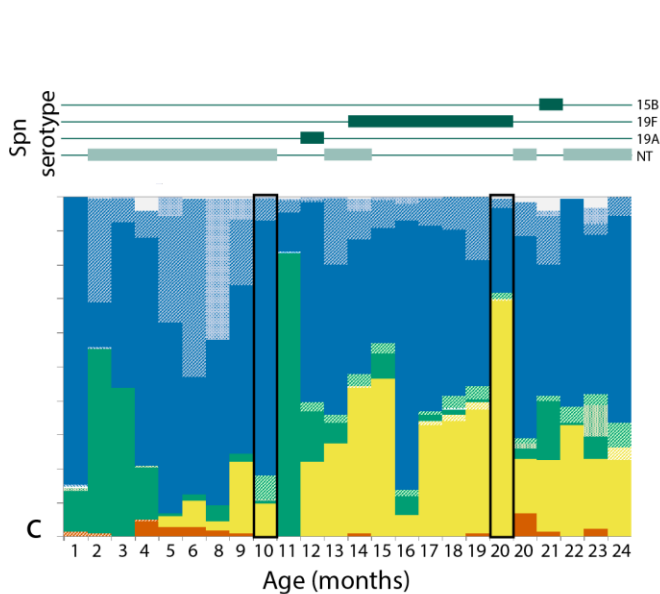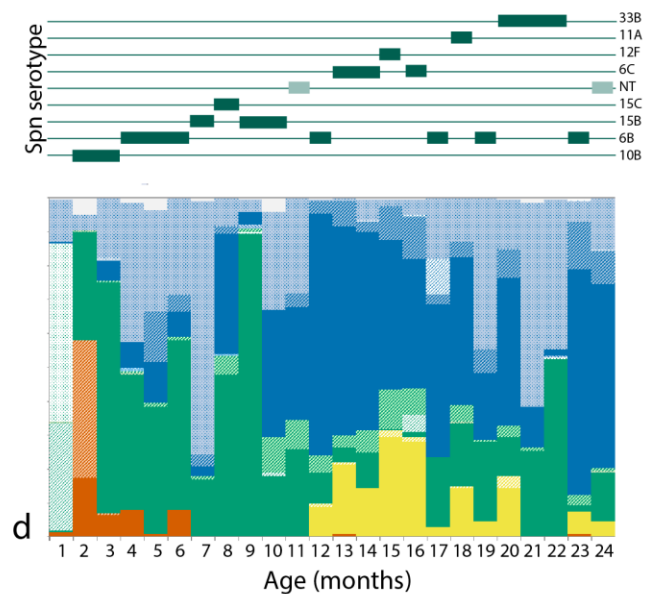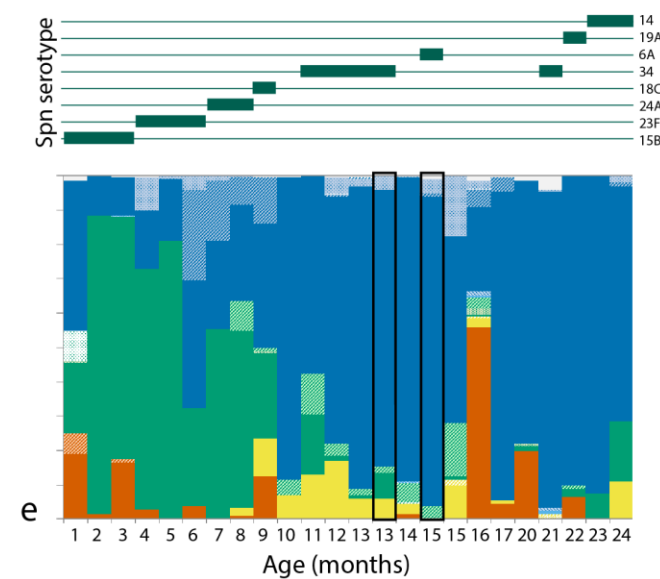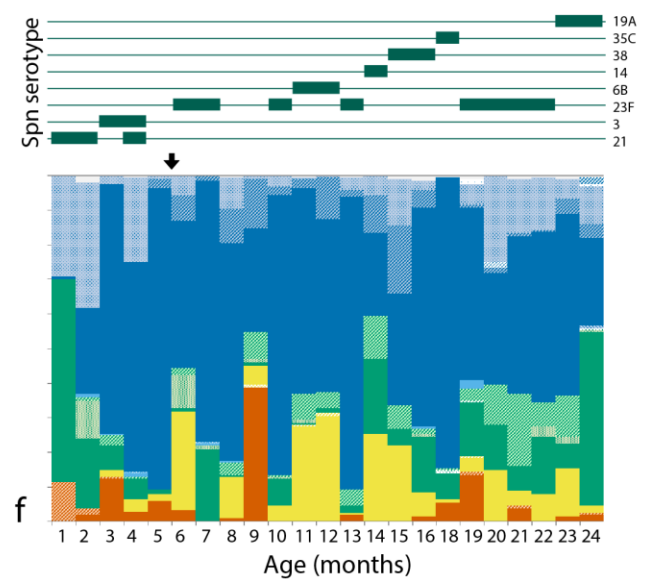

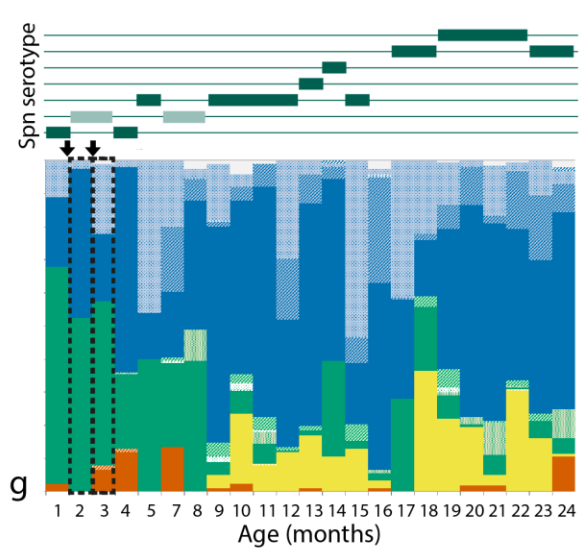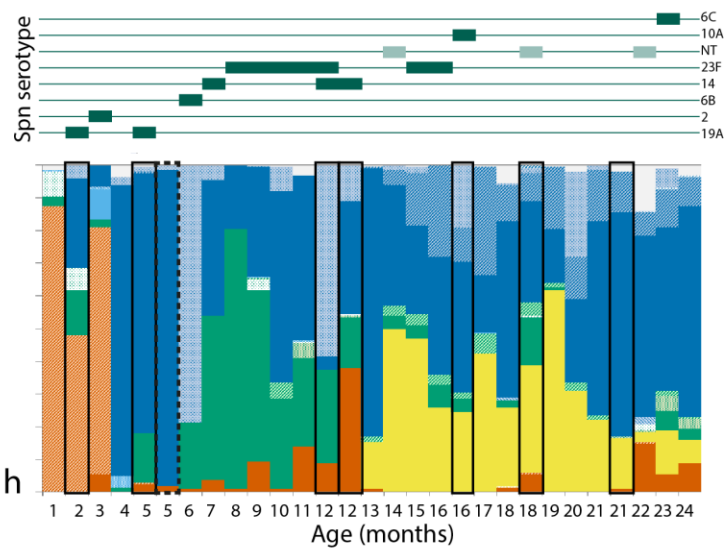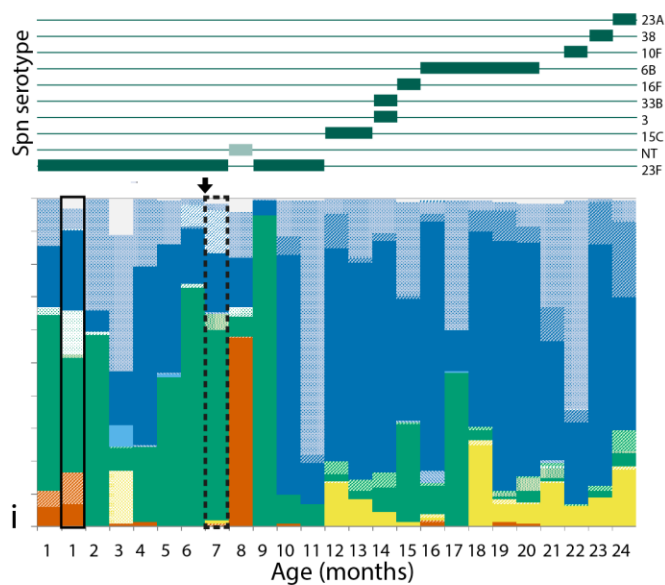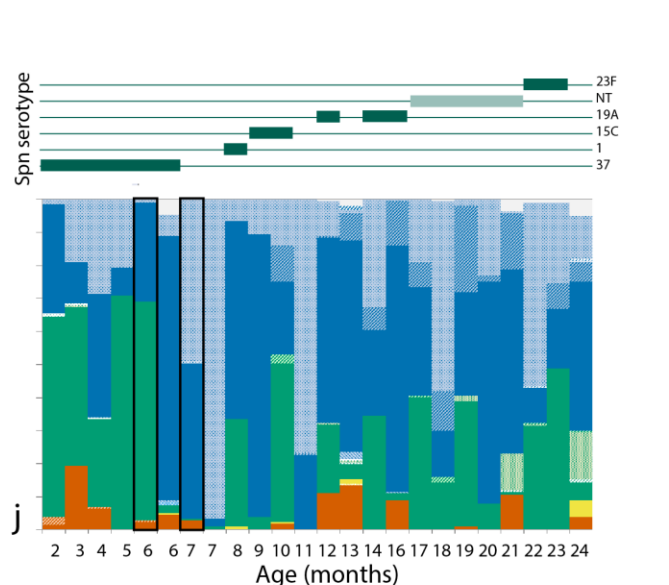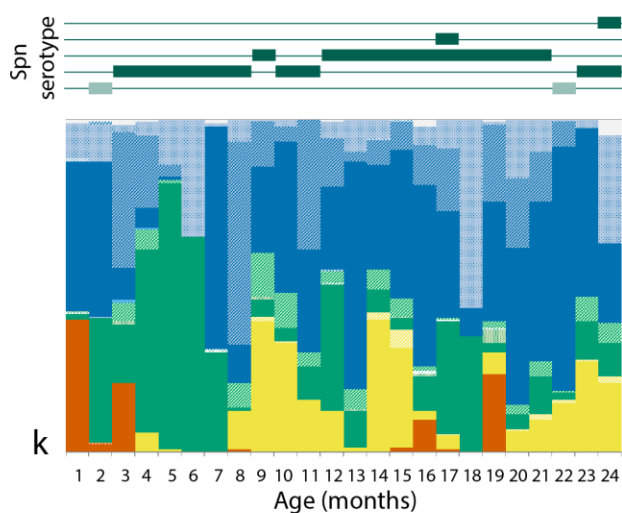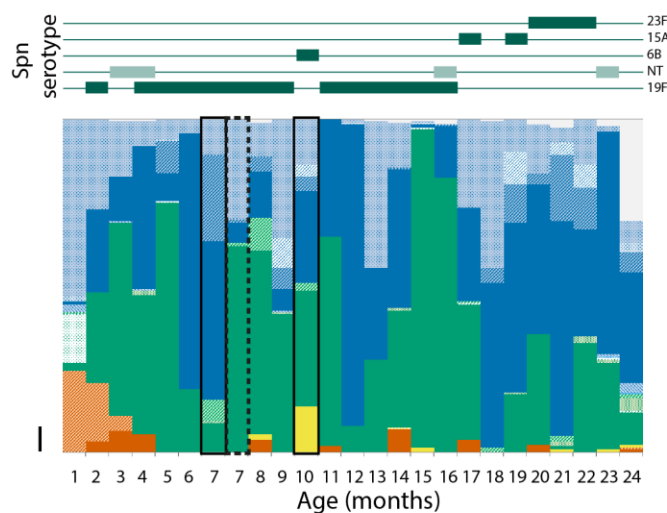

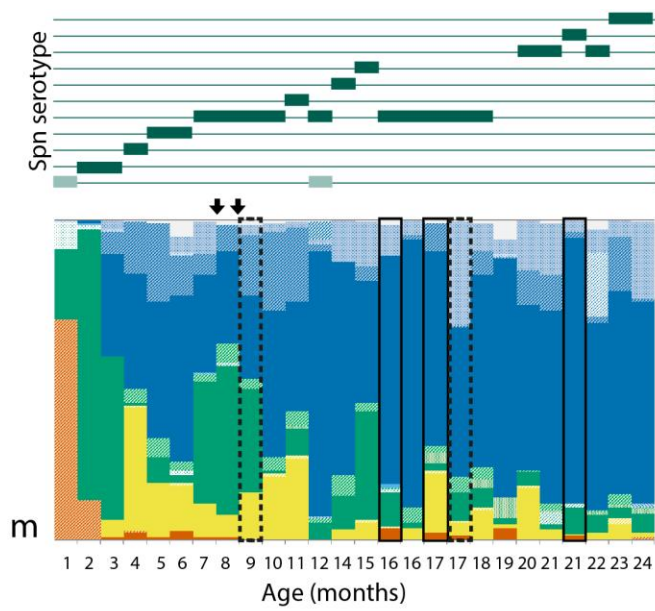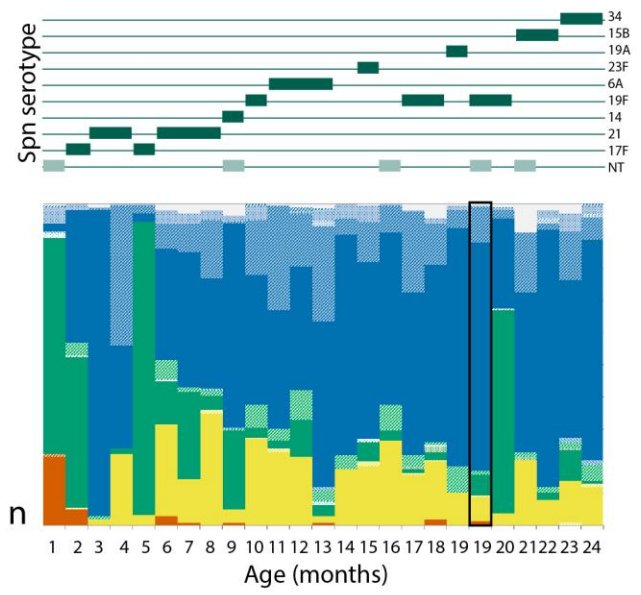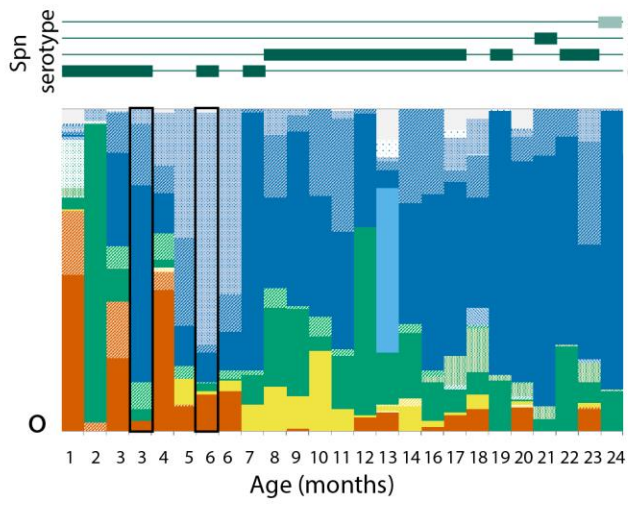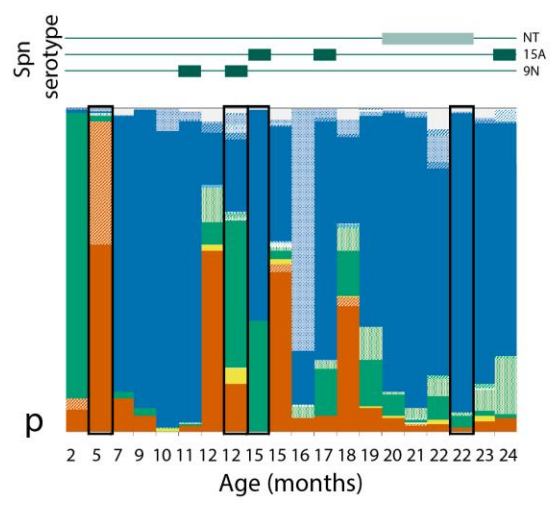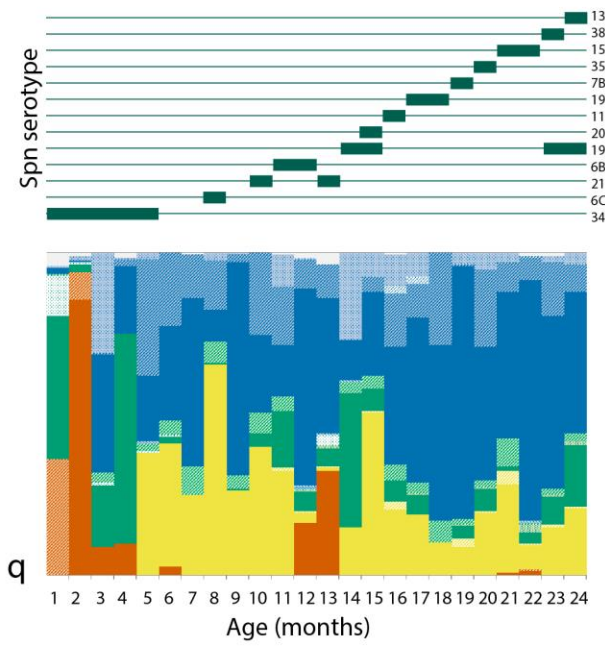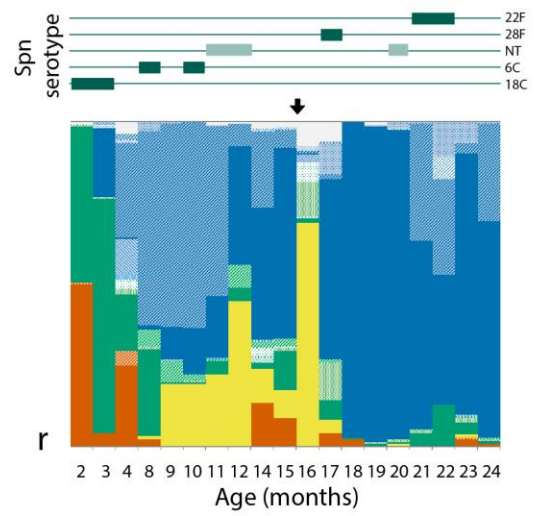

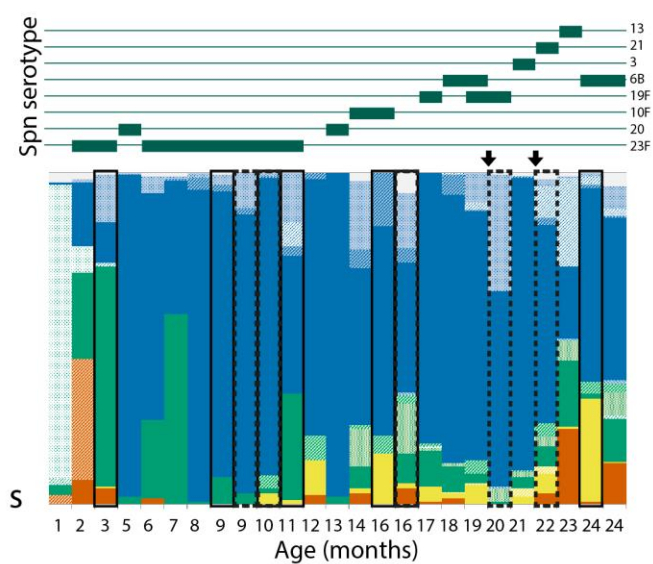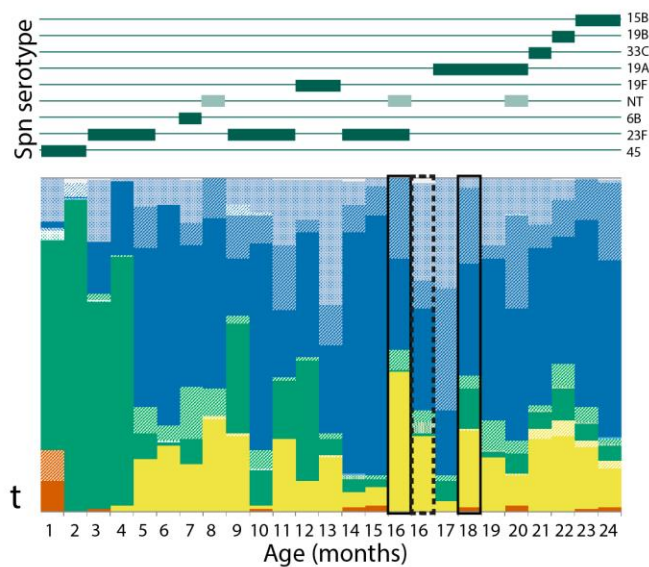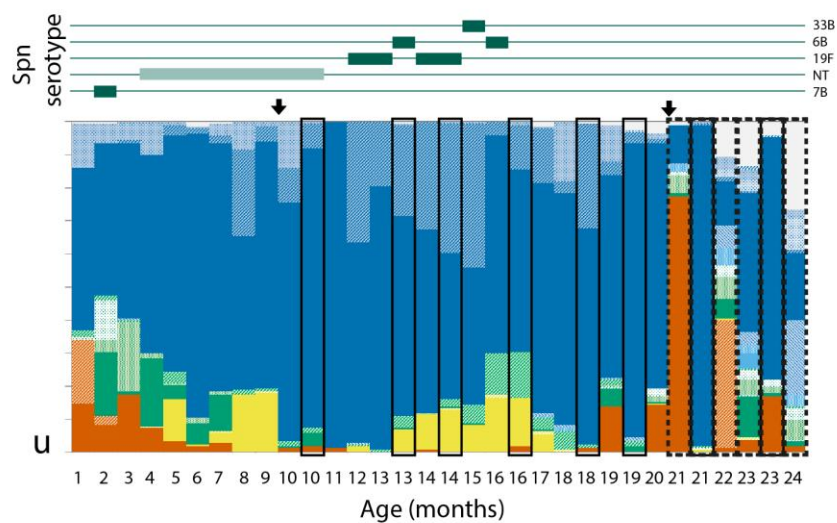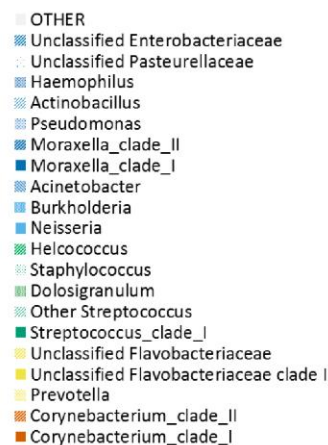

Supplement: S1 Fig — Where cultured, pneumococcal serotype is indicated above each graph. Solid boxes represent swabs taken at the time of ARI immediately preceding amoxicillin treatment, dashed boxes represent swabs taken during antibiotic treatment. Arrows indicate episodes of non-respiratory disease that required antibiotic treatment. 1a) ARI0031, b) ARI0073, c) ARI0106, d) ARI0129, e) ARI0141, f) ARI0179, g) ARI0203, h) ARI0218, i) ARI0237, j) ARI0328, k) ARI0372, l) ARI00450, m) ARI0410 n) ARI9998 (the twin of ARI0410), o) ARI0464, p) ARI0483, q) ARI0484, r) ARI0501, s) ARI0585, t) ARI0604, u) ARI0002 (diagnosed with tuberculosis during the study). (PDF) [file pntd.0005975.s001.pdf]

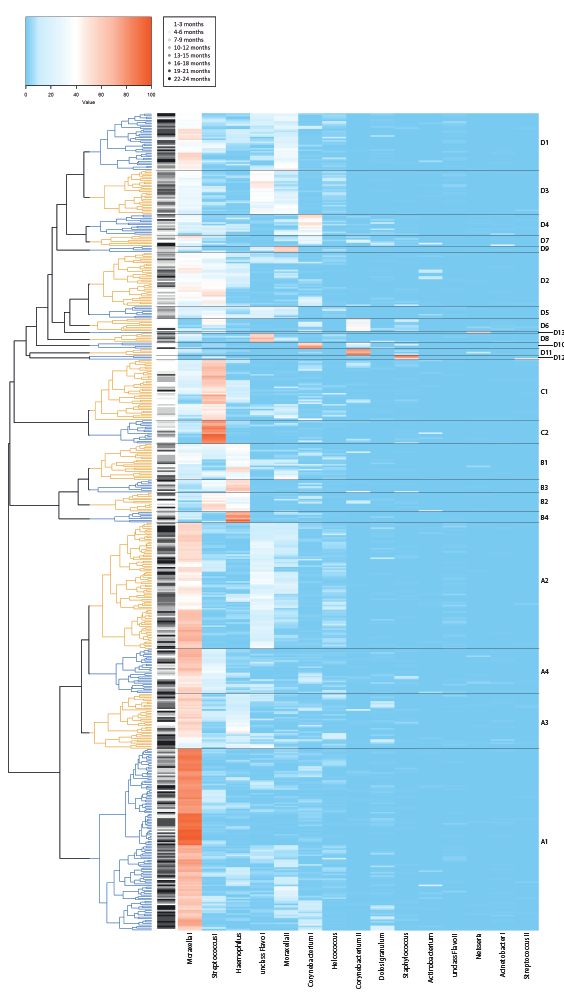

Supplement: S2 Fig — (PNG) [file pntd.0005975.s002.png]

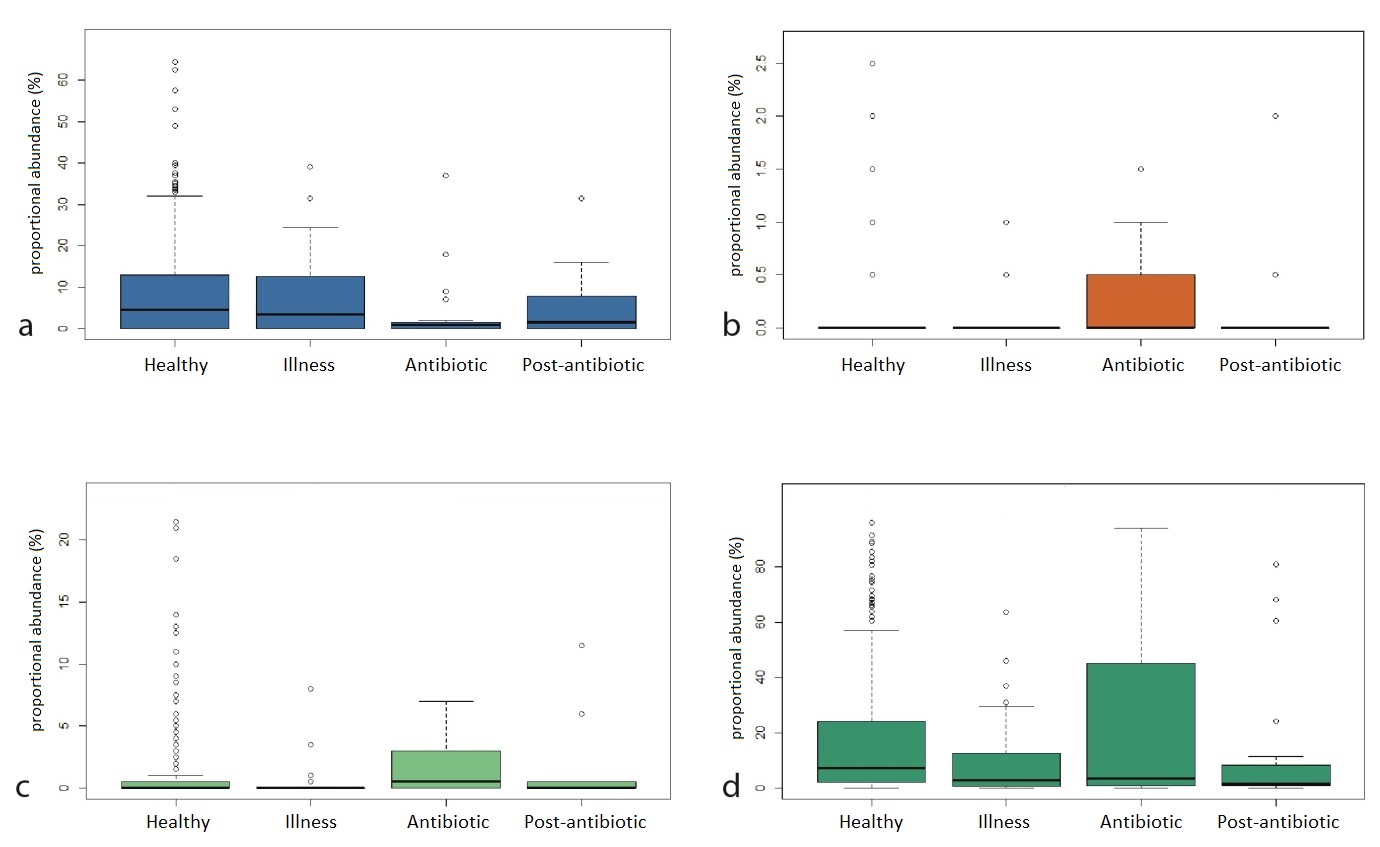

Supplement: S3 Fig — “Healthy”, “antibiotic” and “post-antibiotic” are samples collected at routine monthly intervals, grouped retrospectively based on the concurrent antibiotic consumption. (JPG) [file pntd.0005975.s003.jpg]

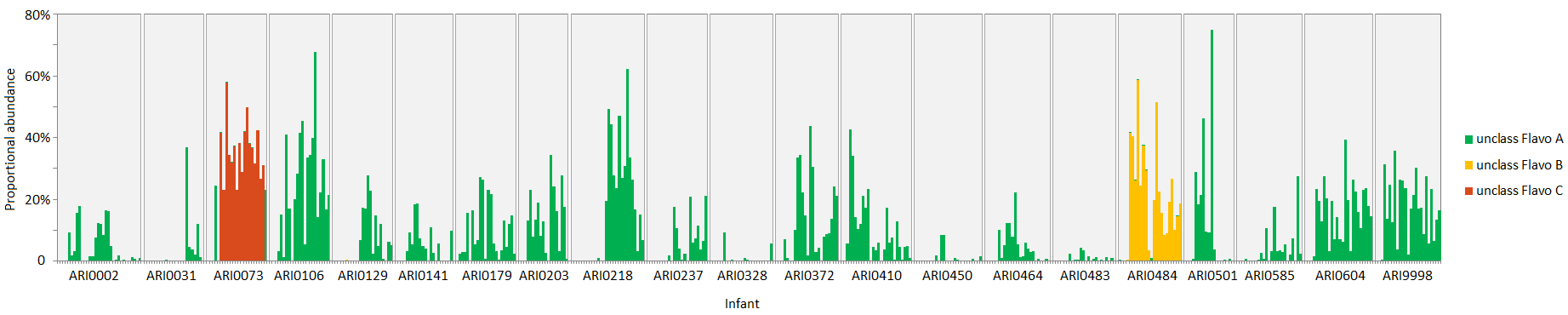

Supplement: S4 Fig — Within an individual the specific oligotype is persistent over time, suggesting that colonisation is stable and transmission is low for this taxon. (PNG) [file pntd.0005975.s004.png]

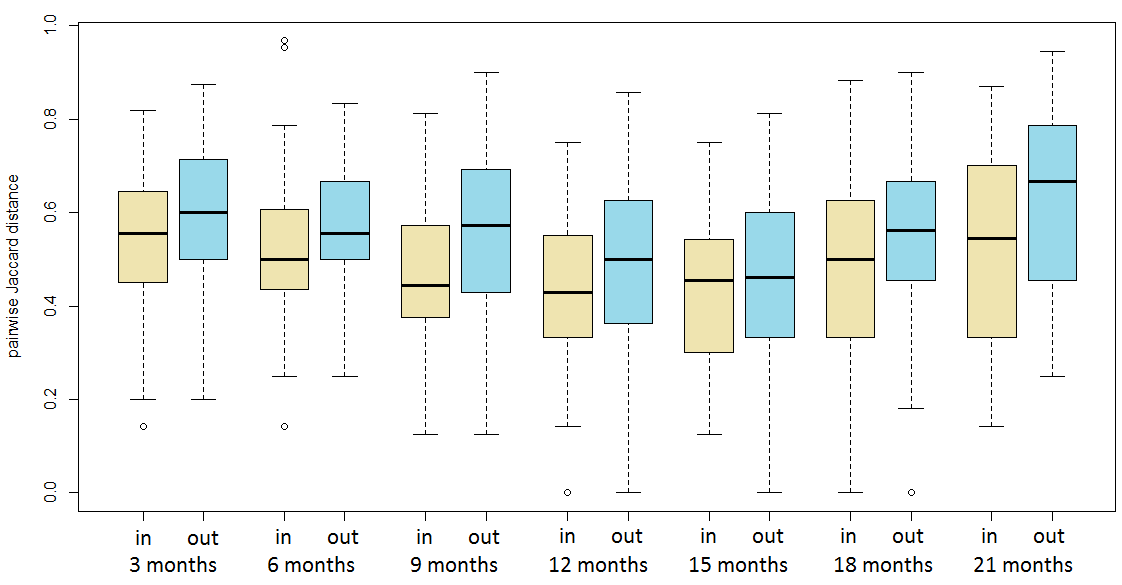

Supplement: S5 Fig — “In” distances are within an individual, comparing one month before/after the timepoint. “Out” distances are between individuals of the same age. Pairwise distances within an individual are lower on average than inter-individual distances at every age, but the difference is not significant. (PNG) [file pntd.0005975.s005.png]

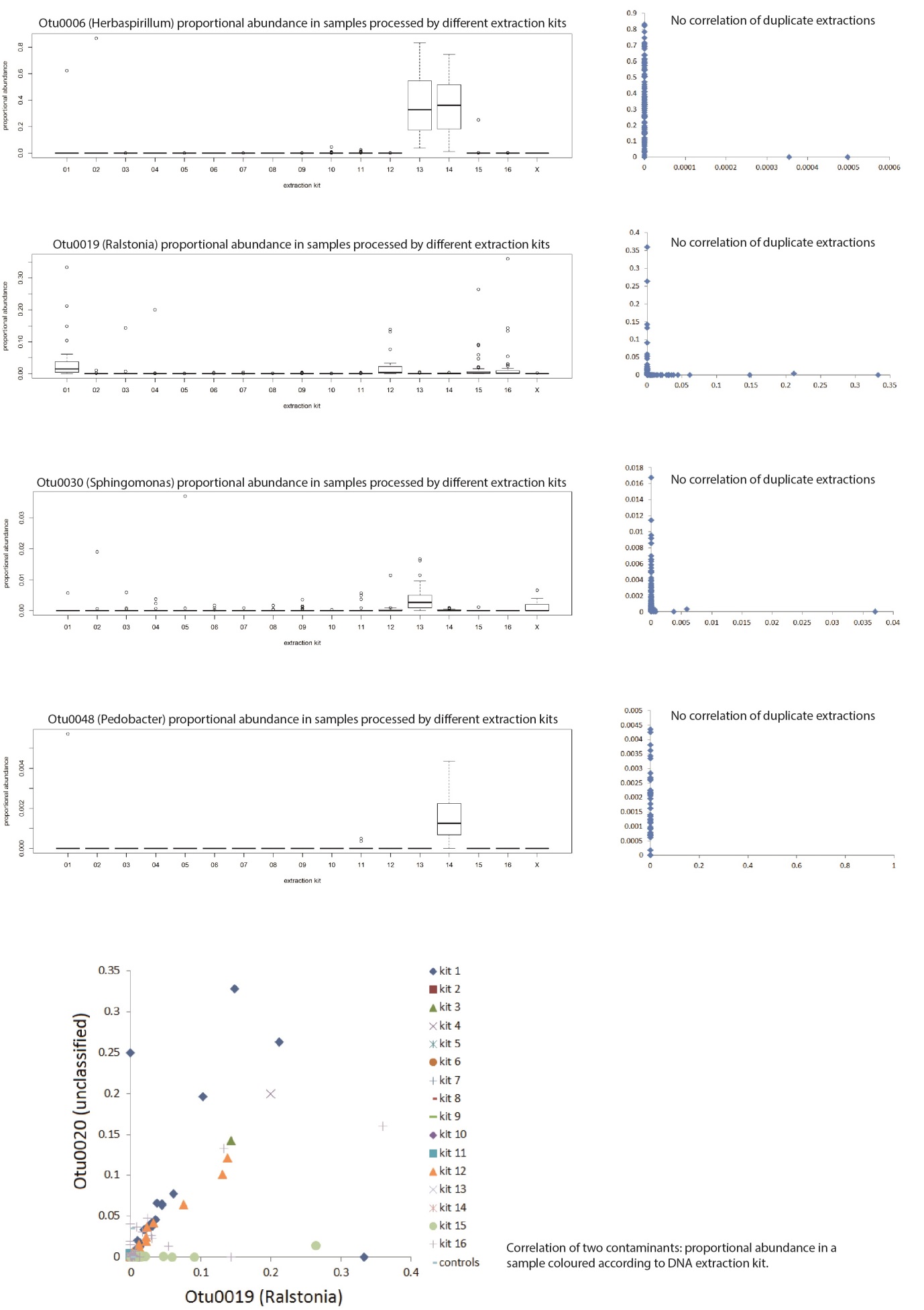

Supplement: S6 Fig — OTUs may be skewed in distribution to specific kits (left side). The DNA introduced during the extraction process will not correlate in duplicate extractions of the same sample (right side). A kit profile may be observed by certain OTUs being associated with one another (bottom): Otu0019 Ralstonia, and Otu0020 an unclassified taxon that has been identified in a variety of published aquatic studies, these correlate well in kits 1 and 12 but Ralstonia is also present independently in kit 15. (JPG) [file pntd.0005975.s006.jpg]
