# Supplementary material for: A longitudinal study of the infant nasopharyngeal microbiota: The effects of age, illness and antibiotic use in a cohort of South East Asian children
Source: PLoS Negl Trop Dis. 2017 Oct 2;11(10):e0005975. doi: 10.1371/journal.pntd.0005975 (PMC5638608; doi:10.1371/journal.pntd.0005975)
Supplement: S1 Methods — (DOCX) [file pntd.0005975.s012.docx]

Supplementary Methods

Subsampling strategy

The depth and consistency of sequencing was not ideal due to technical difficulties in amplicon generation leading to over- and under-representation of some samples in the libraries. We chose to subsample to 200 reads as a compromise between decent sequencing depth and loss of samples, though ideally this rarefaction level would have been significantly higher.

Before choosing 200 reads as an acceptable subsample we compared trends and patterns in the data at different depths and found that a larger number of reads, for example 1,000 per swab, did not make a significant difference to the results but many more swabs then fell below the threshold. At 200 reads, only 5% of samples had to be discarded and the median chao index (observed OTUs/chao estimate) was still 0.947.

For an example of the impact of the different subsampling levels, consider the 27 longitudinal swabs from participant ARI0073 (figure below). The makeup of the microbiota is changed very little by subsampling to 200 reads. Only one swab is lost by subsampling to this level. By subsampling at a higher level such as 1,000 reads, several more swabs are lost which compromises the continuity of sampling.


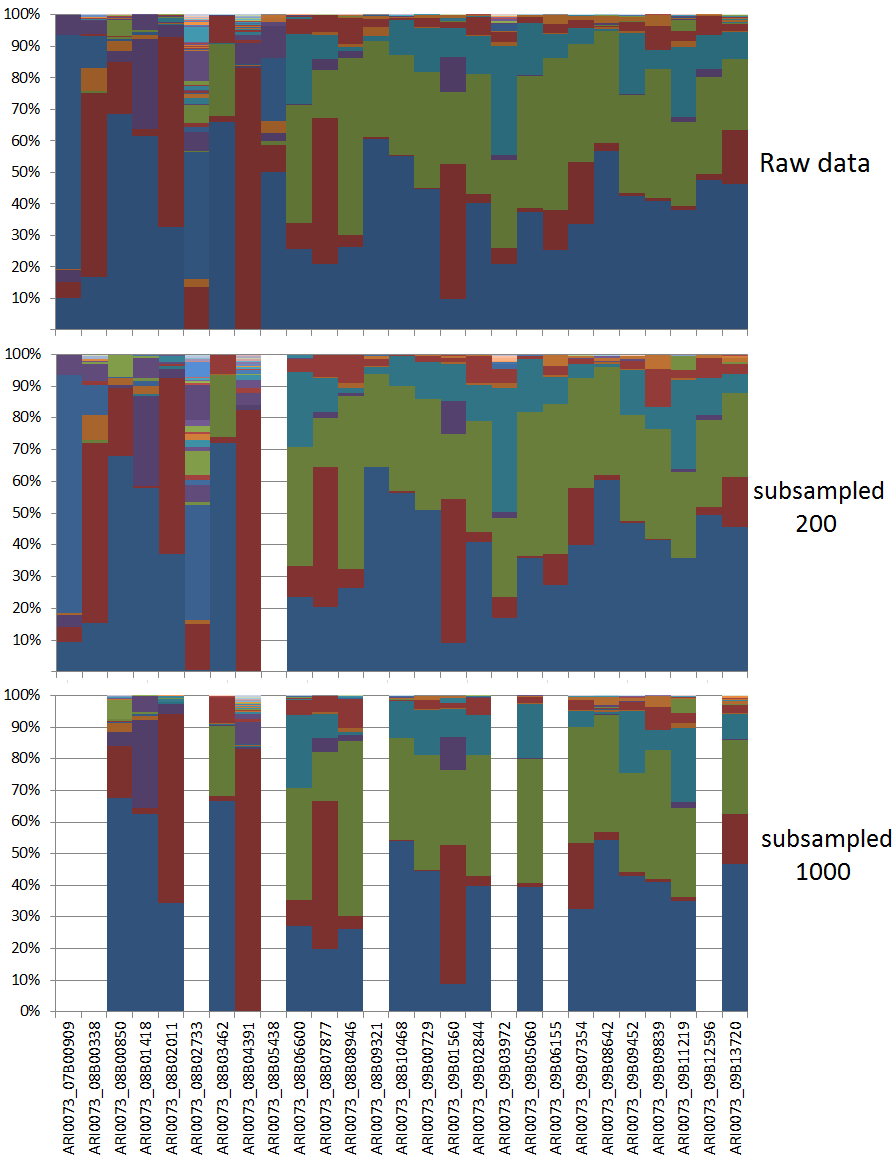


In our dataset just 15 OTUs account for >98% of the aggregated proportional abundance, permitting us to sample less deeply but still observe the key trends. It is supported by the chao index: 46% of samples have an index of 1, and 88% of samples have an index >0.7, meaning that the diversity in most swabs is adequately sampled by 200 reads. This would certainly not be possible with more diverse microbiota samples, such as from faeces, but appears to be sufficient for this study.
