# Supplementary material for: A longitudinal study of the infant nasopharyngeal microbiota: The effects of age, illness and antibiotic use in a cohort of South East Asian children
Source: PLoS Negl Trop Dis. 2017 Oct 2;11(10):e0005975. doi: 10.1371/journal.pntd.0005975 (PMC5638608; doi:10.1371/journal.pntd.0005975)
Supplement: S1 Checklist — (DOCX) [file pntd.0005975.s013.docx]

STROBE Statement—Checklist of items that should be included in reports of ***cohort studies***

|  | Item No | Recommendation |
| --- | --- | --- |
| **Title and abstract** | 1 | (*a*) Indicate the study’s design with a commonly used term in the title or the abstract  [line 2-3] |
|  |  | (*b*) Provide in the abstract an informative and balanced summary of what was done and what was found [line 29-46] |
| Introduction | | |
| Background/rationale | 2 | Explain the scientific background and rationale for the investigation being reported [line 63-68] |
| Objectives | 3 | State specific objectives, including any prespecified hypotheses [line 75-79] |
| Methods | | |
| Study design | 4 | Present key elements of study design early in the paper [line 29-35] |
| Setting | 5 | Describe the setting, locations, and relevant dates, including periods of recruitment, exposure, follow-up, and data collection [line 316-329] |
| Participants | 6 | (*a*) Give the eligibility criteria, and the sources and methods of selection of participants. Describe methods of follow-up [line 316-318, 324-325, 332-333] |
|  |  | (*b*) For matched studies, give matching criteria and number of exposed and unexposed [NA] |
| Variables | 7 | Clearly define all outcomes, exposures, predictors, potential confounders, and effect modifiers. Give diagnostic criteria, if applicable [line 323, 325-329, Table S1, Table S4] |
| Data sources/ measurement | 8* | For each variable of interest, give sources of data and details of methods of assessment (measurement). Describe comparability of assessment methods if there is more than one group [line 323-324, 367-389] |
| Bias | 9 | Describe any efforts to address potential sources of bias [line 392-408] |
| Study size | 10 | Explain how the study size was arrived at [line 324-325] |
| Quantitative variables | 11 | Explain how quantitative variables were handled in the analyses. If applicable, describe which groupings were chosen and why [line 378-389] |
| Statistical methods | 12 | (*a*) Describe all statistical methods, including those used to control for confounding [line 378-384] |
|  |  | (*b*) Describe any methods used to examine subgroups and interactions [line 113-125, 218-222, 262-265] |
|  |  | (*c*) Explain how missing data were addressed [Supplementary Methods] |
|  |  | (*d*) If applicable, explain how loss to follow-up was addressed [NA] |
|  |  | (*e*) Describe any sensitivity analyses [NA] |
| Results | | |
| Participants | 13* | (a) Report numbers of individuals at each stage of study—eg numbers potentially eligible, examined for eligibility, confirmed eligible, included in the study, completing follow-up, and analysed [Table S1, Table S4] |
|  |  | (b) Give reasons for non-participation at each stage [NA] |
|  |  | (c) Consider use of a flow diagram [NA] |
| Descriptive data | 14* | (a) Give characteristics of study participants (eg demographic, clinical, social) and information on exposures and potential confounders [Table S4, line 328-329, |
|  |  | (b) Indicate number of participants with missing data for each variable of interest [Table S1] |
|  |  | (c) Summarise follow-up time (eg, average and total amount) [line 317-319] |
| Outcome data | 15* | Report numbers of outcome events or summary measures over time [NA] |
| Main results | 16 | (*a*) Give unadjusted estimates and, if applicable, confounder-adjusted estimates and their precision (eg, 95% confidence interval). Make clear which confounders were adjusted for and why they were included [NA] |
|  |  | (*b*) Report category boundaries when continuous variables were categorized [age category in Table S1] |
|  |  | (*c*) If relevant, consider translating estimates of relative risk into absolute risk for a meaningful time period [NA] |
| Other analyses | 17 | Report other analyses done—eg analyses of subgroups and interactions, and sensitivity analyses [line 113-125, 162-171, 218-222, 262-265] |
| Discussion | | |
| Key results | 18 | Summarise key results with reference to study objectives [line 281-293] |
| Limitations | 19 | Discuss limitations of the study, taking into account sources of potential bias or imprecision. Discuss both direction and magnitude of any potential bias [line 204-209, 232-238, 255-256, 289-293] |
| Interpretation | 20 | Give a cautious overall interpretation of results considering objectives, limitations, multiplicity of analyses, results from similar studies, and other relevant evidence [line 281-311] |
| Generalisability | 21 | Discuss the generalisability (external validity) of the study results [line 304-311] |
| Other information | | |
| Funding | 22 | Give the source of funding and the role of the funders for the present study and, if applicable, for the original study on which the present article is based [in funding statement]. |

*Give information separately for exposed and unexposed groups.

**Note:** An Explanation and Elaboration article discusses each checklist item and gives methodological background and published examples of transparent reporting. The STROBE checklist is best used in conjunction with this article (freely available on the Web sites of PLoS Medicine at http://www.plosmedicine.org/, Annals of Internal Medicine at http://www.annals.org/, and Epidemiology at http://www.epidem.com/). Information on the STROBE Initiative is available at http://www.strobe-statement.org.
